# Speed Controls in Translating Secretory Proteins in Eukaryotes - an Evolutionary Perspective

**DOI:** 10.1371/journal.pcbi.1003294

**Published:** 2014-01-02

**Authors:** Shelly Mahlab, Michal Linial

**Affiliations:** 1 School of Computer Science and Engineering, The Hebrew University of Jerusalem, Jerusalem, Israel; 2 Department of Biological Chemistry, Institute of Life Sciences, Sudarsky Center for Computational Biology, The Hebrew University of Jerusalem, Jerusalem, Israel; Weizmann Institute of Science, Israel

## Abstract

Protein translation is the most expensive operation in dividing cells from bacteria to humans. Therefore, managing the speed and allocation of resources is subject to tight control. From bacteria to humans, clusters of relatively rare tRNA codons at the N′-terminal of mRNAs have been implicated in attenuating the process of ribosome allocation, and consequently the translation rate in a broad range of organisms. The current interpretation of “slow” tRNA codons does not distinguish between protein translations mediated by free- or endoplasmic reticulum (ER)-bound ribosomes. We demonstrate that proteins translated by free- or ER-bound ribosomes exhibit different overall properties in terms of their translation efficiency and speed in yeast, fly, plant, worm, bovine and human. We note that only secreted or membranous proteins with a Signal peptide (SP) are specified by segments of “slow” tRNA at the N′-terminal, followed by abundant codons that are considered “fast.” Such profiles apply to 3100 proteins of the human proteome that are composed of secreted and signal peptide (SP)-assisted membranous proteins. Remarkably, the bulks of the proteins (12,000), or membranous proteins lacking SP (3400), do not have such a pattern. Alternation of “fast” and “slow” codons was found also in proteins that translocate to mitochondria through transit peptides (TP). The differential clusters of tRNA adapted codons is not restricted to the N′-terminal of transcripts. Specifically, Glycosylphosphatidylinositol (GPI)-anchored proteins are unified by clusters of low adapted tRNAs codons at the C′-termini. Furthermore, selection of amino acids types and specific codons was shown as the driving force which establishes the translation demands for the secretory proteome. We postulate that “hard-coded” signals within the secretory proteome assist the steps of protein maturation and folding. Specifically, “speed control” signals for delaying the translation of a nascent protein fulfill the co- and post-translational stages such as membrane translocation, proteins processing and folding.

## Author Summary

Measurements of translation by ribosomal profiling and additional large-scale methods support the notion that the elongation speed and ribosomal occupancy are tightly regulated. We revisited the proteomes of a number of organisms, from yeast to human, and focused on the appearance of codons' clusters that impact the speed of translation elongation. Thus, transcripts are analyzed according to their encoded “traffic signs.” Specifically, translation by free- or endoplasmic reticulum (ER)-bound ribosomes differs substantially with respect to the codon clusters' distribution at the beginning of the coding region. Discretization of all transcripts to consecutive segments exposed the uniqueness of secreted and membranous proteins that have a signal peptide (SP). Similarly, a non-random codon distribution characterized proteins with “targeting peptides” for mitochondria and for GPI-anchor, while the bulk of the proteome carry no significant pattern of their codons. We conclude that translation via an ER co-translocation process imposes unique constraints on translation efficiency that match with the fate of the proteins as secreted, membranous, mitochondrial-targeted or GPI-anchored. Tuning the translation of a nascent protein is essential for coping with the constraints imposed by membrane-bound translation for a successful ER translocation and protein processing for maturation and folding.

## Introduction

In dividing cells, the process of translation elongation consumes most of the cell energy and resources [1]–[3]. The rate of translation must be tightly controlled for coping with the cell demands and its limited resources. Specifically, translation efficiency is determined by the amount of proteins that are produced from the coding mRNA. In a more mechanistic view, translation efficiency is reflected by the preferable allocation of ribosomes on the mRNA [4]. Sequence-based features such as mRNA folding energy, positioning of individual amino acids (AAs) and codons govern the translation efficiency [5]–[7]. Failure in coordinating the ribosomal flow leads to ribosomal drop-off [3], translation errors [8], frame-shift [9] and protein misfolding [10]. Direct measurements of ribosome density from *in vivo* studies confirmed that translational rates differ between transcripts [11]. Moreover, the rate may vary by several folds on the same mRNA [2], [12], [13].

Several factors govern protein translation rate and accuracy (see discussion in [3], [14], [15]). A dominant parameter in dictating translation rate is the nature of the codons at the initial segment of the transcripts [16]. Other features include the competition on ribosome binding [17], mRNA folding energy [5], accessibility of specific tRNAs [18] and CG content [5]. A dominating parameter of translation efficiency from E. coli to human is the codon usage [19], [20]. The coding usage of a broad range of organisms positively correlated with cellular proteins' expression levels and thus, indirectly, with translation efficiency [21], [22].

In all eukaryotes, the decoding of mRNAs to proteins obeys the same rules [23]. The genomic tRNA copy number (CN) strongly correlates with the needs for intracellular tRNA levels [24]. This property is best captured by the tRNA adaptation index (tAI) [19] that balances between the decoding rules and the tRNA CN [25]. Indeed, in humans, tAI appropriates the actual abundance of tAI in healthy and diseased cells [26].

In eukaryotes, a distinction should be made between proteins that are translated by the soluble, cytosolic ribosome (CYTO-Rb) and the membrane-bound ribosomes (MEM-Rb). The latter cover the proteins destined to the secretory systems (endoplasmic reticulum (ER), Golgi, endosomes, lysosomes, plasma membrane and the extracellular space) [27]. A common feature of the secretory proteins is the presence of signal peptide (SP) at the N′-terminal [28]. Alternatively, membranous proteins that lack SP (*e.g.*, many G-protein coupled receptors) use their first TMD as a membrane signal. Translation of the secretory proteins at the ER membranes is a multiphase process that is based on coordinated steps of translation, translocation and folding [13], [29], [30].

In this study, we hypothesized that proteins of CYTO-Rb and MEM-Rb translation differ in their translation elongation management. A local tRNA adaptation pattern at the N′-terminal which starts with segments of lowly adapted tRNAs, followed by segments of highly adapted tRNAs, is characteristic of secreted and membranous SP-proteins but not identified in the bulk of the proteins or in other regions of the transcripts. Such patterns are shared by a large number of eukaryotic proteomes and found also in proteins that are designated to the mitochondria. The impact of “traffic signs” on the management of translating ER-bound ribosomes is discussed in view of recent experimental evidence on translation rates.

## Results

### Translation elongation efficiency is approximated by tRNA adaptation index

**Figure 1:**
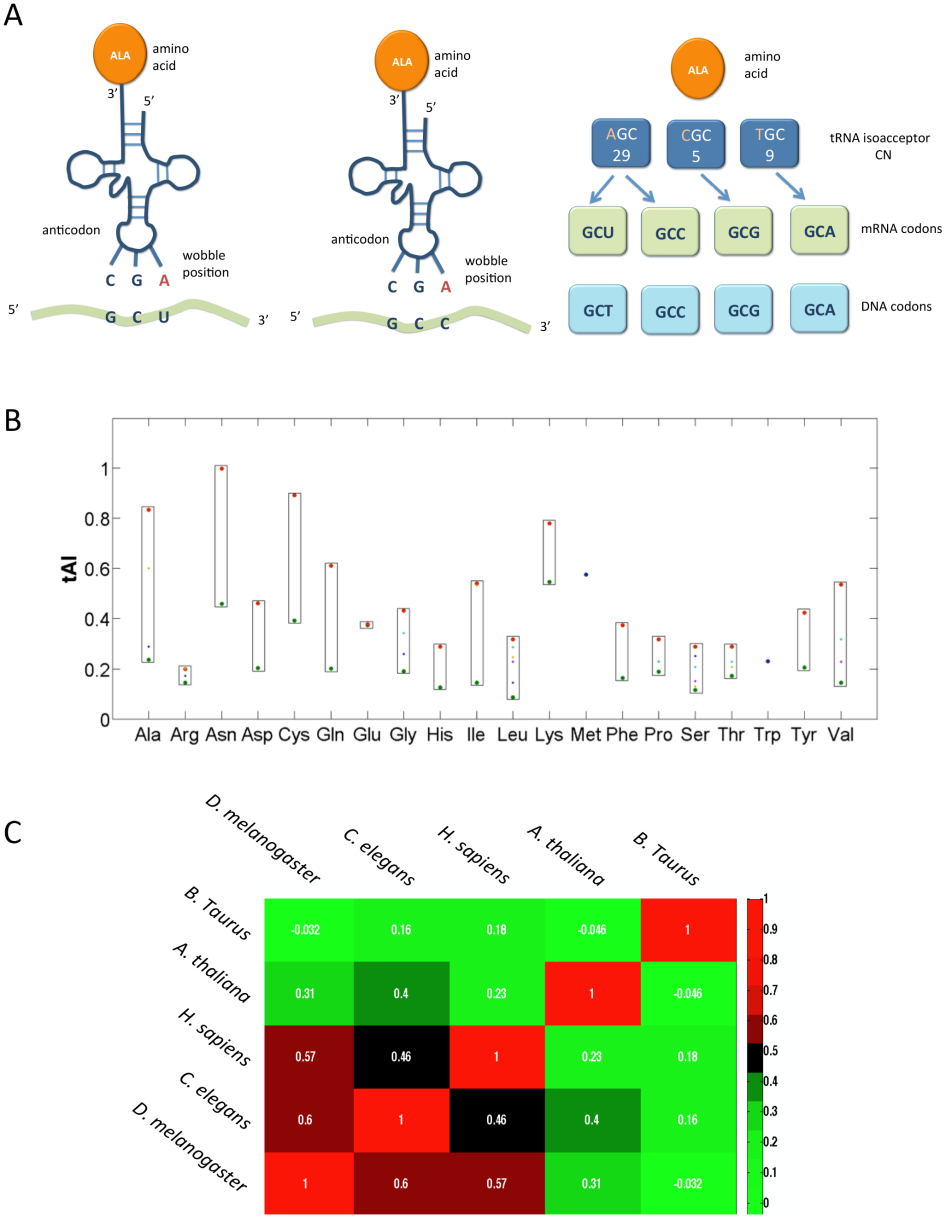
tRNA isoacceptors and adaptation index. (**A**) Illustration of the decoding by tRNA. The alanine (Ala) charged tRNAs that recognize GCU and GCC belong to the same isoacceptor. Decoding is performed according to the wobble rules [73]. Alanine (Ala) is decoded by three groups of isoacceptor tRNAs. The genomic tRNA copy number (CN) from H. sapiens is marked. Specifically, the number of genes for Ala is 43 (the sum of the CN of all isoacceptor groups). Codons are always read by the 5′ to 3′ directionality from DNA or mRNA. (**B**) The range of codon tAI that can be assigned to each AA in H. sapiens is shown. Codon tAI is determined by the CN of tRNAs for that codon and according to the coupling of tRNA at the wobble position. The tAI for each codon is marked by a colored dot. Tryptophan (Trp) and Methionine (Met) are encoded by a single codon. For the other AAs a range of tAI values are shown according to the number of codons (2, 3, 4 and 6 codons). Note that Arg, Ser and Leu that are decoded by 6 codons each, do not necessarily have a wide range of tAI values. The minimal and maximal tAI values for each of the AAs are colored green and red, respectively. (**C**) Clustering of multicellular model organisms by the correlation calculated according to a vector of the tAI values (61 codons). The Spearman correlation coefficient between each pair of species is color-coded. The tAI codon values for each organism is listed in Table S1.

An estimation of the effect of the tRNA abundance on the efficiency of the translation is captured by the tRNA adaptation index (tAI) (See Materials and Methods). The pairing of tRNA with the mRNAs is not unique in the case of the Wobble pairing (Figure 1A). Each organism differs by the number and the relative appearance of tRNA isoacceptors for decoding the 20 amino acids (AAs, 61 codons). Synonymous codons are associated with a broad range of tAI values (Figure 1B). Some AAs (e.g., Arginine) are encoded by 6 codons but the range of their tAI values is still very narrow. On the other hand, a broad range of tAI values is associated with AAs that have only two codons each (e.g., Asparagine and Cysteine) (Figure 1B).

The tRNAs copy number (CN) is subjected to evolutionary forces and thus differs substantially throughout the evolutionary tree. For example, there are 287 tRNA genes in the budding yeast *S. cerevisiae* but as many as 3790 tRNA genes in *Bos Taurus*. The tAI value that is assigned to each codon varies substantially among different organisms. While the correlations among human, D. melanogaster, C. elegans are moderate, the correlations with *B. taurus* or *A. thaliana* (flowering plant) are negligible (Figure 1C). The tAI codon values for each organism is listed in Table S1.

### Translation efficiency marks are encoded in the human secretory proteome

The translation of proteins in eukaryotes is executed in two settings: Proteins that are translated by free ribosomes (coined cytoplasmic ribosome, CYTO-Rb) and ER bound ribosomes (coined membranous ribosome, MEM-Rb). We partitioned the entire proteomes into four non-overlapping groups (Table S2):

- Signal Peptide (SP) proteins that are not located at the membrane (SP not TMD). These are mostly secreted proteins (e.g., hormone peptides, growth factors).
- SP proteins with TMD. These are proteins that contain at least one TMD but are translocated to the ER via an SP recognition mode. Additional step leads to a protein maturation following the removal of the SP (e.g., HLA class I histocompatibility antigens, Cadherins).
- Integral membrane proteins that lack SP (TMD not SP). The initial TMD is used for insertion of the protein to the translocation pore (i.e., translocon). The topology of these proteins is determined by the presence of a stop signal along the sequence. The first TMD serves as an anchor signal.
- Proteins that lack SP or TMD and are translated by free ribosomes (CYTO-Rb, simply refer to as “Cytosolic”). Recall that the final destination of these proteins may not be restricted to the cytosol (*e.g,* nuclear proteins).

**Figure 2:**
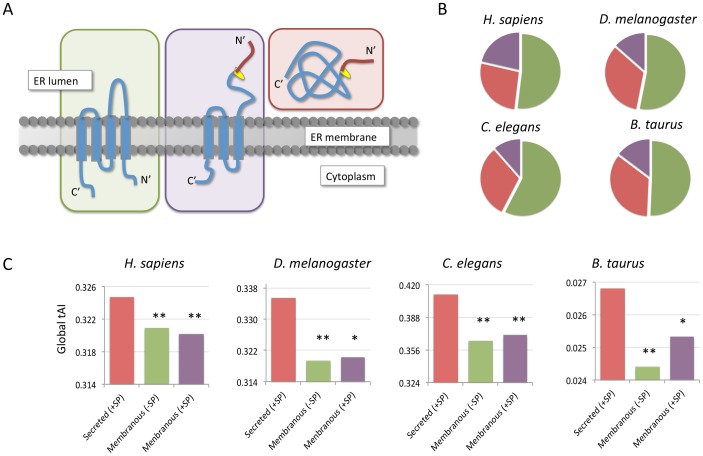
The secretory proteome. (**A**) Partition of the secretory proteome with respect to membrane topologies is shown. The secreted proteins (red background) contain a Signal peptide (SP, red string) that is cleaved in the ER lumen. The site for cleavage by the SP protease is colored yellow. The membranous fraction is divided according to the presence (purple background) or absence (green background) of SP. All the three groups are translated by MEM-Rb. (**B**) Pie diagrams show the partition of the secretory proteome: (i) Proteins that have TMD but lack of SP sequence (TMD non-SP), (ii) Proteins that have SP but each protein has one or more TMD (SP and TMD) and (iii) Secreted proteins with SP in their N′-terminus (marked in red, SP non-TMD). The rest of the proteins are soluble proteins that are translated by CYTO-Rb. The majority of the secretory proteome in all the 4 model organisms - human (*H. sapiens*), fly (*D. melanogaster*), worm (*C. elegans*) and bovine (*B. taurus*) are membranous proteins without SP (green). For these proteins, ER translocation is mediated via internal TMDs. For the detailed number of proteins in each organism see Table S2. (**C**) Average global tAI values for each groups of the secretory proteome as in (B). The histograms show analysis of the entire secretory proteomes from *H. sapiens, D. melanogaster*, *C. elegans* and *B. taurus*. Similar trends apply for Yeast (*S. cerevisiae*) and plant (*A. thaliana*). The statistical significance is based on the p-value calculated from the Kolmogorov–Smirnov (KS) test. The statistical significance are marked by asterisks. With p-values E-5 to E-10 (*) and <E-10 (**), (for detailed statistical analysis see Table 1).

Groups (i–iii) compose the secretory proteome (Figure 2A). The human proteome consists of 18,434 proteins. Among them 26% include at least one TMD and an additional 9.5% are secreted proteins that contain SP. A similar partition is reported for fly, worm and bovine (Figure 2B) and other model organisms. The tAI of each coding sequence is computed (see Materials and Methods), and the average “global tAI” for the analyzed proteins' group was defined (see Materials and Methods). Each of the three protein groups that together compose the secretory proteome displays a distinct global tAI (Figure 2C). For example, the p-value of the human secreted proteins (marked as “SP-not TMD” group) relative to membranous proteins without SP (TMD not SP) is 2.58e-11. The calculated p-values of the secreted proteins with respect to membranous proteins with SP (TMD and SP) and the cytosolic group are 1.08e-14 and 9.01e-12, respectively.

Comparing the average global tAI values for the secretory and cytosolic protein groups in different organisms is shown in Table 1. The main observation (Figure 2C) demonstrates that secreted proteins that have SP tend to have higher global tAI relative to the proteins of the membranous groups (TMD, with or without SP). While the absolute values of the global tAI are different for each organism (based on codon tAI, Table S1), the trend of low tAI for the membranous proteins relative to the secreted proteins is surprisingly robust (Figure 2C). We extended the analysis to include also yeast and plant representatives. The average values of the calculated global tAI values for (i) cytosolic proteins, (ii) SP-no TMD (iii) SP and TMD and (iv) TMD not SP are listed in Table S3.

**Table 1:** Statistical KS tests for the global tAI values that were calculated for 6 model organisms' proteomes.

Organism	Groups^a^	TMD non-SP	SP and TMD	Cytosolic
*H. sapiens*	SP non-TMD	**2.58e-11**	**1.08e-14**	**9.01e-12**
	TMD non-SP		0.0374	4.84e-4
	SP and TMD			**2.18e-5**
*B. taurus*	SP non-TMD	**3.96e-34**	**8.11e-9**	9.18e-3
	TMD non-SP		9.61e-4	**7.42e-85**
	SP and TMD			**2.22e-15**
*D. melanogaster*	SP non-TMD	**8.33e-10**	**5.36e-6**	3.3e-4
	TMD non-SP		0.488	**7.19e-29**
	SP and TMD			**8.82e-11**
*C. elegans*	SP non-TMD	**7.79e-22**	**5.01e-11**	0.012
	TMD non-SP		2.31e-4	**2.84e-57**
	SP and TMD			**1.22e-13**
*S. cerevisiae*	SP non-TMD	**6.48e-23**	**1.67e-12**	**3.3e-16**
	TMD non-SP		0.476	**1.14e-11**
	SP and TMD			6.6e-3
*A. thaliana*	SP non-TMD	**1.12e-17**	**1.24e-30**	**1.34e-6**
	TMD non-SP		**3.28e-13**	**2.67e-10**
	SP and TMD			**2.77e-28**

^a^Partition of the proteomes to 4 exclusive groups is according to UniProtKB annotations for TMD and SP. Statistical significance <1.0e-5 is shown in bold.

We show the statistical significance among each pair of the protein groups for 6 organisms (Table 1). The statistical difference between the two exclusive sets of membranous proteins (with/without SP) is minimal (with p-value>1.0e-4, Table 1). For example, the p-values of the global tAI values for the yeast-secreted proteins relative to other groups range from 1.67e-12 to 6.48e-23 (Table 1). A striking observation is that secreted proteins and the soluble fraction (i.e., CYTO-Rb translation) specify high average global tAI values with regard to the membranous proteins. A similar trend was observed in all six tested organisms (included yeast and flowering plant, Table S3).

### Global tAI correlates with mRNA expression levels and protein abundance

**Figure 3:**
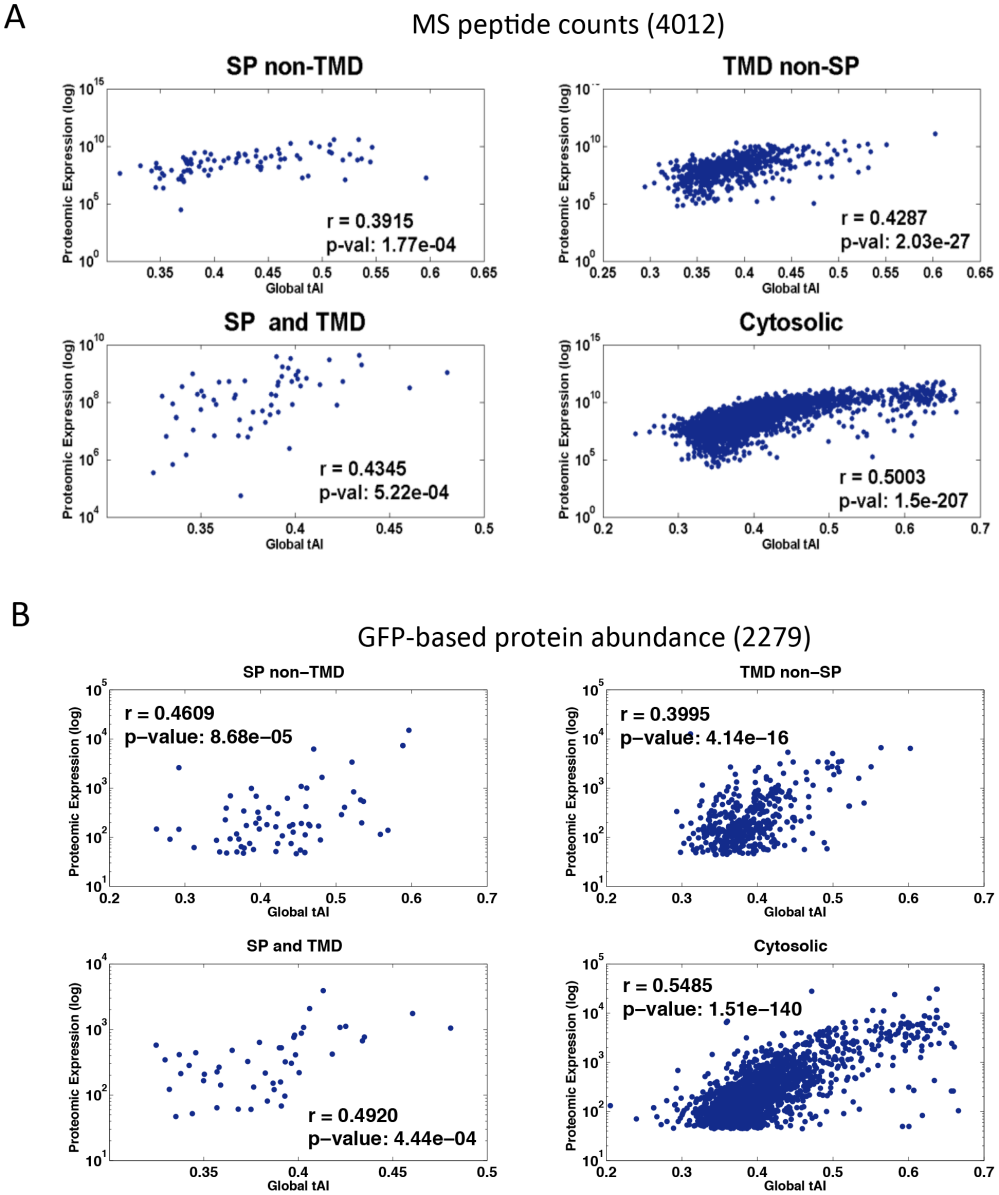
Correlation between yeast protein abundance and global tAI. (**A**) Mass spectrometry (MS) data were from the yeast quantitative proteome [33]. Protein abundance is measured from the match of the MS peptide-spectrum. Each spectrum is associated with a peptide that is re-assigned to its parent protein. The analysis covered 4012 proteins divided as follows: SP non-TMD: 87; TMD non-SP: 582; SP and TMD: 60; Cytosolic: 3283. (**B**) Quantitative proteomics [34] was measured by estimating the fluorescence from the tagged-GFP. The analysis covered 2279 proteins divided as follows: SP non-TMD: 67; TMD non-SP: 383; SP and TMD: 47; Cytosolic: 1782. The protein abundance and the global tAI are plotted and the correlation coefficient (r) and the p-values are indicated.

Many determinants govern the protein abundance in eukaryotic cells [11]. The contribution of sequence-dependent determinants to the rates of translation and degradation has been estimated [31]. A positive correlation between the gene tAI and its expression was determined from the signature of gene expression microarrays [32]. We tested whether the average higher global tAI that was associated with the secreted (SP non-TMD) and the cytosolic proteins (Table S3) relative to membranous proteins reflects a difference in the expression levels. We took advantage of the experiments with high coverage of the yeast proteome and compared the protein abundance and the global tAI. We used a resource from mass spectrometry (MS) peptide counts [33] (total of 4012 proteins, Figure 3A) and the quantitative data from GFP-tagged proteins [34] (total of 2279 proteins, Figure 3B). We found substantial agreement between the results from these complementary technologies (compare 3A and 3B). The strongest correlation was noted between the global tAI values and the cytosolic proteins. However, the significance of the correlation between the global tAI and the proteins of the secreted proteome is rather weak (SP not TMD). We suggest that the relatively high global tAI is associated with an overall expression level for the majority of the proteins that are translated by free ribosomes (i.e., accounts for 78% and 81% of the analyzed proteins, Figure 3A and 3B, respectively). However, a high expression level is not supported for the secreted protein group. Additional parameters such as protein length, AA usage and CG content were also tested. The length of the proteins from the group “SP and TMD” was significantly longer than the rest of the proteins (P value = 1e-4). But the secreted proteins group (SP not TMD) and the “TMD not SP” group that differs in their tAI (Figure 2C) have no difference in protein length (p value = 0.133). All other correlations show a borderline statistical significance. We concluded that the tAI is strongly associated with protein abundance only for the cytosolic proteins. The same trend was found for the human proteome (data analyzed from [35]).

### A robust signal at the N′-terminal specifies the secreted proteome

**Figure 4:**
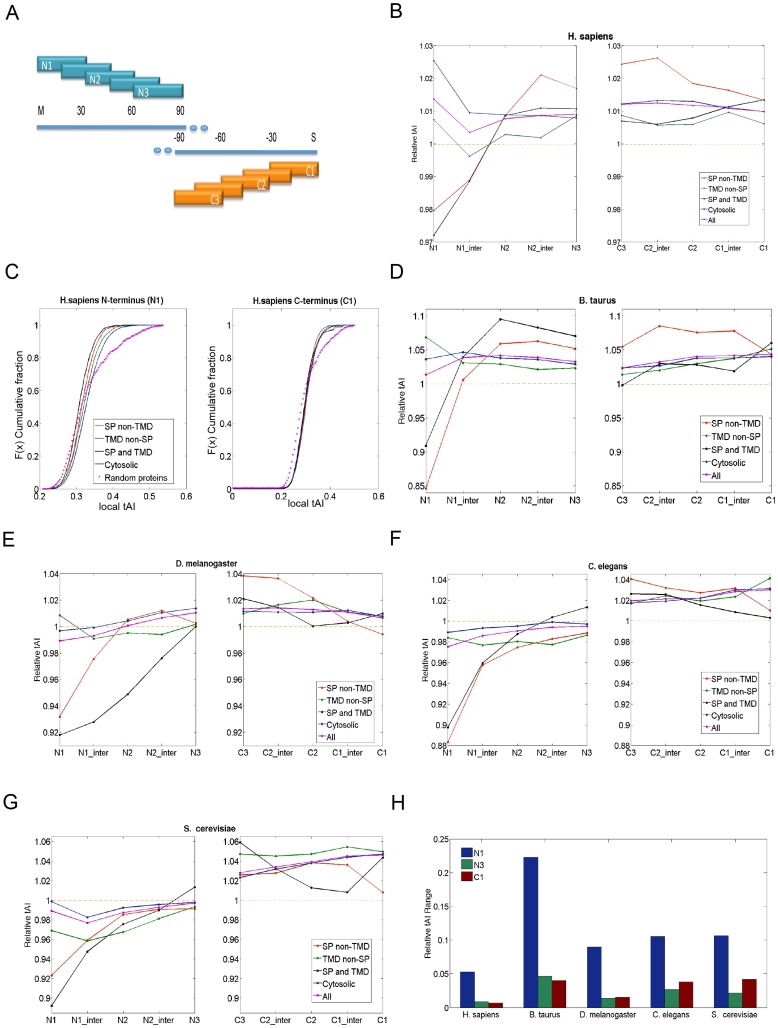
Analysis of local tAI profiles. (**A**) A schematic description of the 5 segments, each for 30 codons from the N′-terminal region and the C′-terminal of the coding sequence. (**B**) Relative tAI profile of the N′- and C′-terminal segments of the human proteome according to 4 group partition (as in Table S2). Each of the protein group is color coded as follows: Red, SP non-TMD; Black, SP and TMD; Green, TMD non-SP; Blue, Cytosolic proteins. Purple, the entire proteome, marked as “All.” Pink asterisks, the random proteins according to length distribution of the proteome. (**C**) Cumulative distribution of proteins according to the tAI values of the N1 and C1 segments. The data are based on all tAI values that were compiled in (B) for N′- and C′-termini. Note that for the N′-terminal but not the C′-terminal, the cumulative distribution of each of the four protein groups is distinctive. The statistic of the cumulative distribution for human proteome is shown in Table 2. Relative tAI profile for *B. taurus* (**D**), *D. melanogaster* (**E**), *C. elegans* (**F**) and *S. cerevisiae* (**G**). (**H**) The range of relative tAI values of N1, N3, and C1 segments of all tested organisms. The relative tAI range is defined as the highest averaged relative tAI subtracted by the lowest averaged relative tAI value among the four protein groups within the same segment.

The secreted proteins showed significantly higher global tAI values (Figure 2C, Table S3). We tested the possibility that the tested protein groups may carry segmental information in addition to their global tAI values. To analyze the segmental properties of the proteomes, we discretized the transcripts to segments of 30 codons. The same notations were applied for the C′-terminus, starting from the last codon of the protein (Figure 4A). The results are presented as “Relative tAI,” which is defined as the current segments' tAI divided by the calculated value of the global tAI of the coding sequence. This measure allows comparing the trends among organisms. Using the Relative tAI values (and not the absolute tAI values) cancels out the inherent difference in expression levels that are associated with the tested proteins groups (Figures 2–3).

Among the analyzed model organisms, the annotations for the human proteome are accurate and complete. According to the four groups partition (Figure 2B and the cytosolic fraction), the SP-containing proteins are characterized by an occurrence of lowly adapted tRNAs segment (coined LATS) at the N′-terminal (∼45 codons) followed by highly adapted tRNAs (HATS) (Figure 4B). Notably, proteins that contain SP with or without TMD display a similar profile. All protein groups converged at segment N3 (codons position 60–90, Figure 4B). It is important to note that the “Relative tAI” profile of the entire proteome (combined all 4 groups, marked “All”, Figure 3B) shows no outstanding position-based pattern. Additional segments (*e.g.*, N4) provided no additional information and will not be discussed further.

Figure 4C shows the cumulative distribution of tAI values for each of the analyzed protein groups for N1 and C1 segments from a human proteome. The statistical difference between the N1 and C1 segments is significant (Table 2). Actually, both the N1 and the C1 segments differ significantly from a random selection of a 30-codon segment (Kolmogorov-Smirnov (KS) test, Figure 3C, Table 2). The calculated p-values versus the random sets range between 1.0e-15 to 1.0e-22 for N1, and 1.0e-12 to 1.0e-27 for C1. More importantly, the statistical tests show significant p-values (7.6e-6 to 2.1e-57) for the characteristics of the N1 segment among the four protein groups, while the p-values for the C1 segments are statistically insignificant (Table 2).

**Table 2:** Statistical differences (KS test) between segments of tAI values for partition of the human proteome and randomized sequences.

	SP non-TMD	TMD non-SP	SP and TMD	Cytosolic	Random
**SP non-TMDa**	1	7.57e-06	1.25e-07	**2.77e-28**	**3.83e-20**
**TMD non-SP**	0.000722	1	**6.83e-23**	**3.49e-13**	**3.98e-15**
**SP and TMD**	0.001162	0.149803	1	**2.08e-57**	**1.60e-22**
**Cytosolic**	0.030616	0.004063	0.007944	1	**1.79e-22**
**Random**	**1.33e-22**	**8.41e-23**	**1.11e-12**	**4.47e-27**	1

Upper and lower triangles are based on 30-codon segments identified as N1 and C1, respectively. Statistical significance <1.0e10 is shown in bold.

The tAI segmental analysis was extended to other model organisms including *B. taurus* (Figure 4D), *D. melanogaster* (Figure 4E), *C. elegans* (Figure 4F) and *S. cerevisiae* (Figure 4G). Assessing the significance of the differences in the “Relative tAI” values for the different segments of the four protein groups is achieved by comparing the maximal range of the computed average relative tAI among the four groups. For example, the “Average Relative tAI'”of the N1 in *H. sapiens* spans as much as 0.053 while the C1 deviates by only 0.007. We demonstrated these range differences of N1, N3 and C1 segments for all the tested organisms (Figure 4H). A similar pattern is generalized and the range of “Average Relative tAI” of N1 is significantly higher than that of N3 or C1. In this view, the range in values of segment N3 is considered a statistical noise.

As many of the secreted proteins (e.g., hormones, growth factors) are short proteins, we tested the effect of protein length on the observed segmental tAI profile. We confirmed that the impact of the protein length of the segmental local tAI is negligible. Specifically, we partitioned the SP-proteins to very short (90–240 AAs) and very long (>1,000 AAs) protein groups. We found that the trend of the tAI profiles is insensitive to the length. The “very short” and “very long” proteins originated from the same distribution (t-test, p-value = 0.72).

**Figure 5:**
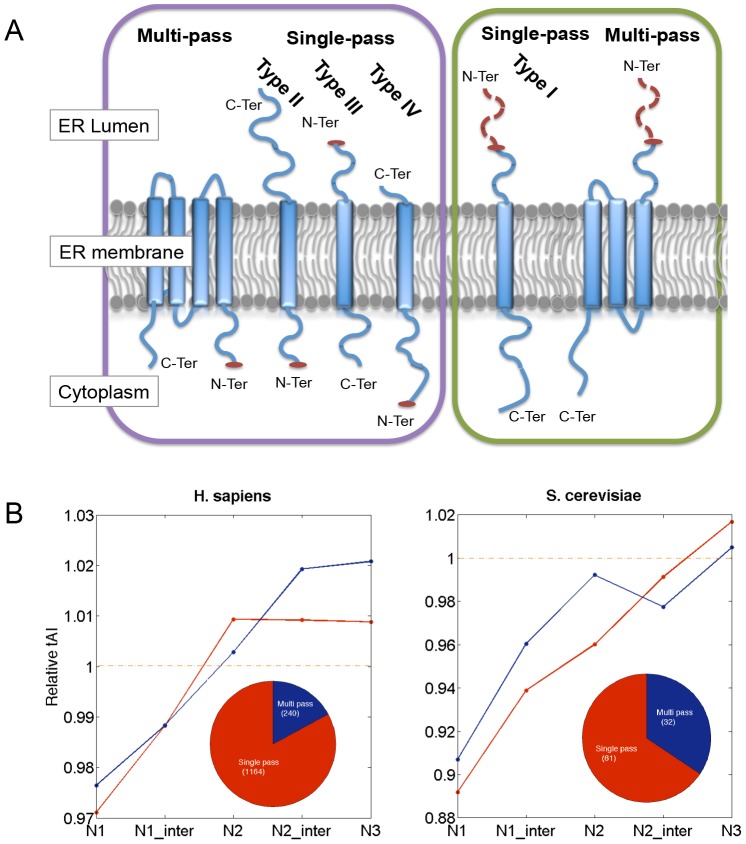
Analysis of membranous proteins according to their topologies. (**A**) Partition of membranous proteins to single or multi-pass proteins. The set is composed from two protein groups (Table S2): (i) Proteins that have TMD but lack the SP sequence (TMD not-SP), (ii) Proteins that have SP but each protein has one or more TMD (SP and TMD). The protein groups are separated according to the topologies as single TMD or multiple TMDs (marked as Type I–IV). (**B**) Relative tAI analysis according of the membrane topologies. The profile of the N′-terminal is shown (N1 to N3, see Figure 3A) for *H. sapiens* and *S. cerevisiae*.

We tested the differential tAI segmental profiles of membranous proteins (composed of the groups of “SP and TMD” and “TMD not SP”) according to the separation to single (marked as types I–IV) and multi-pass proteins (Figure 5A). This type of partition tests whether the membrane topology governs the characteristics of the tAI segmental profile (shown in Figures 4B–4G). It is evident that the existing of SP dominates the profile irrespectively to the number of TMDs or the protein topology within the membrane (Figure 5B). The analysis is limited to yeast and humans due to the poor annotations on membranous protein topologies for the other model organisms.

Alignment of the proteins at their N′- and C′-terminal segments was essential to reveal the signal for the SP-proteins, irrespective of the membrane topology of a specific protein (Figure 5). For membranous proteins that lack SP, the first TMD acts as the anchor signal. We further tested whether a codon dependent signal is encoded in the TMD. To this end, we aligned all sequences from the “TMD not SP” group by their first TMD (Figure S1). We found that the segmental tAI values of the first TMD differs from the observation of the SP-proteins. Actually, the “anchored TMD” shares no local tAI characteristics. We concluded that it is not the hydrophobicity per se that dictates the local tAI properties but instead, the SP sequences are characterized by clusters of lower adapted codons followed by clusters of highly adapted segments.

### Generalizing speed controls toward organelle destination and subcellular localization

The robust phenomena of differential codon usage according to their tAI property along the transcript is not restricted to the N′-terminal segment. The Glycosylphosphatidyl inositol (GPI) anchored proteins reach the ER through an SP dependent process. For these proteins, an additional modification occurs following a proteolytic cleavage at a C′-terminal peptide of the nascent peptide [36]. We tested whether a signal for GPI lipid anchoring is encoded by segmental tAI measurements.

**Figure 6:**
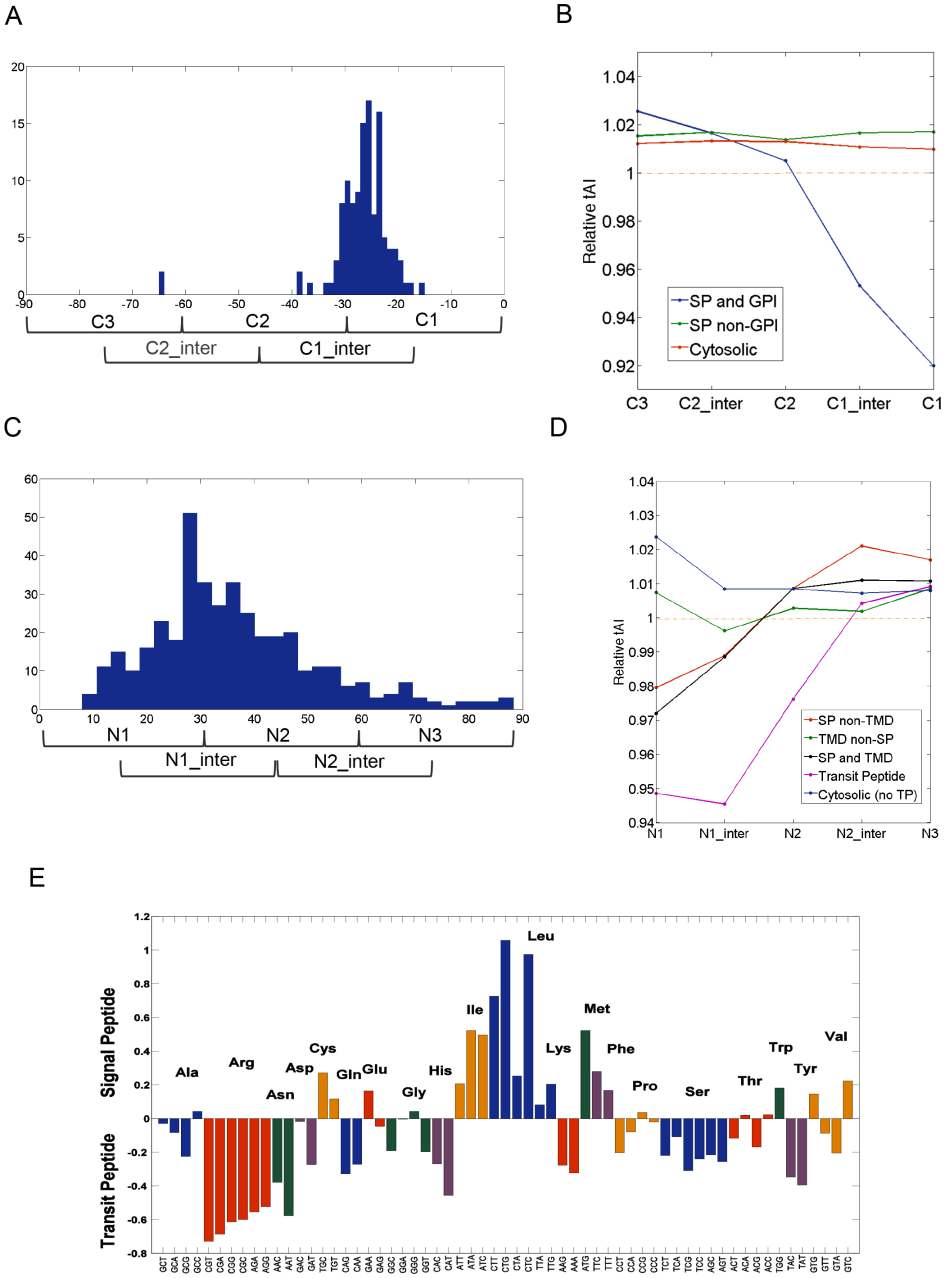
Analysis of the local segmental tAI profiles for GPI-anchored and Transit peptide (TP)-proteins. (**A**) Histogram of the cleavage site relative to the end of the coding transcript for GPI-anchored proteins. Length is measured relative to the stop codon. (**B**) Relative tAI profile at C′-terminal segments for 128 human GPI-anchored proteins at the C′-terminal region. (**C**) Histogram of the cleavage site relative to the initiator Methionine for the TP-proteins. (**D**) Relative tAI profile of 499 human TP proteins at the N′-terminal region. (**E**) Relative codon usage in SP- and TP-proteins. Y-axis scale is the relative codon usage in SP sequences divided by the relative codon usage in the TP sequences. Codons that belong to the same AA are colored as a group.

We separated the proteins that are predicted as GPI-anchor proteins [37]. Figure 6A shows a histogram for the cleavage site with respect to the last codon (marked as codon 0). In the majority of the cases, the cleavage sites are positioned within the C1 segment (codon marked as -25). The average segmental tAI profile for the 128 human GPI-proteins is shown (Figure 6B). Remarkably, the AAs composition of the GPI-anchor proteins is poorly conserved. Still, the GPI-anchor proteins are characterized by the significance of LATS at their final segment (C1, ∼30 codons, Figure 6B). Thus, GPI-anchor proteins are marked by evolutionary signals at both, the N′- and C′-termini.

As opposed to the previously mentioned cases of GPI-anchored and SP-proteins that are modified at the ER on the nascent chain, translocation of mitochondrial proteins occurs as a post-translational stage. Hundreds of proteins reach the different compartments of the mitochondria (and chloroplasts in plants) by sophisticated mechanisms [38], [39]. Many of these mitochondrial targeted proteins have a cleavable Transit Peptide (TP) in their N′-terminals. There are 499 proteins annotated to have TP in humans. Figure 6C shows the cleavage sites with respect to the initiator Methionine. For the majority of the proteins, the cleavage sites are positioned within the N1 or the N1-intermediate segments. The similarity of the local segmental tAI to the profile of the SP-proteins is evident (Figure 6D). TP adopts a more extreme value (“Relative tAI” of 0.95 in *H. sapiens*) for an extended segment relative to the SP-proteins (Figure 6D).

An overlap in the segmental profiles for the SP and TP protein is striking. Figure 6E demonstrates that when the AA compositions of the SP and the TP are compared, the overlap in the AAs usage is minimal. These results postulate as to the generality of the phenomenon. Notably, the marked difference in codon usage of the SP and TP segments argues for an unrestricted selection that supports a pattern of LATS followed by HATS. Such a design may be used as a general trend for management of protein targeting to sub-cellular compartments and organelles.

### The profile at the N′-terminal segments is determined by preferred selection of codons

A key sequence feature of the SP is the central helical region that is dominated by Leu and Ala with some occurrence of Val, Phe and Ile. We show that the SP proteins have a preferable use of some amino acids (e.g., Leu and Trp), but a limited use of Asn, Asp, Ser, Thr and Arg.

**Figure 7:**
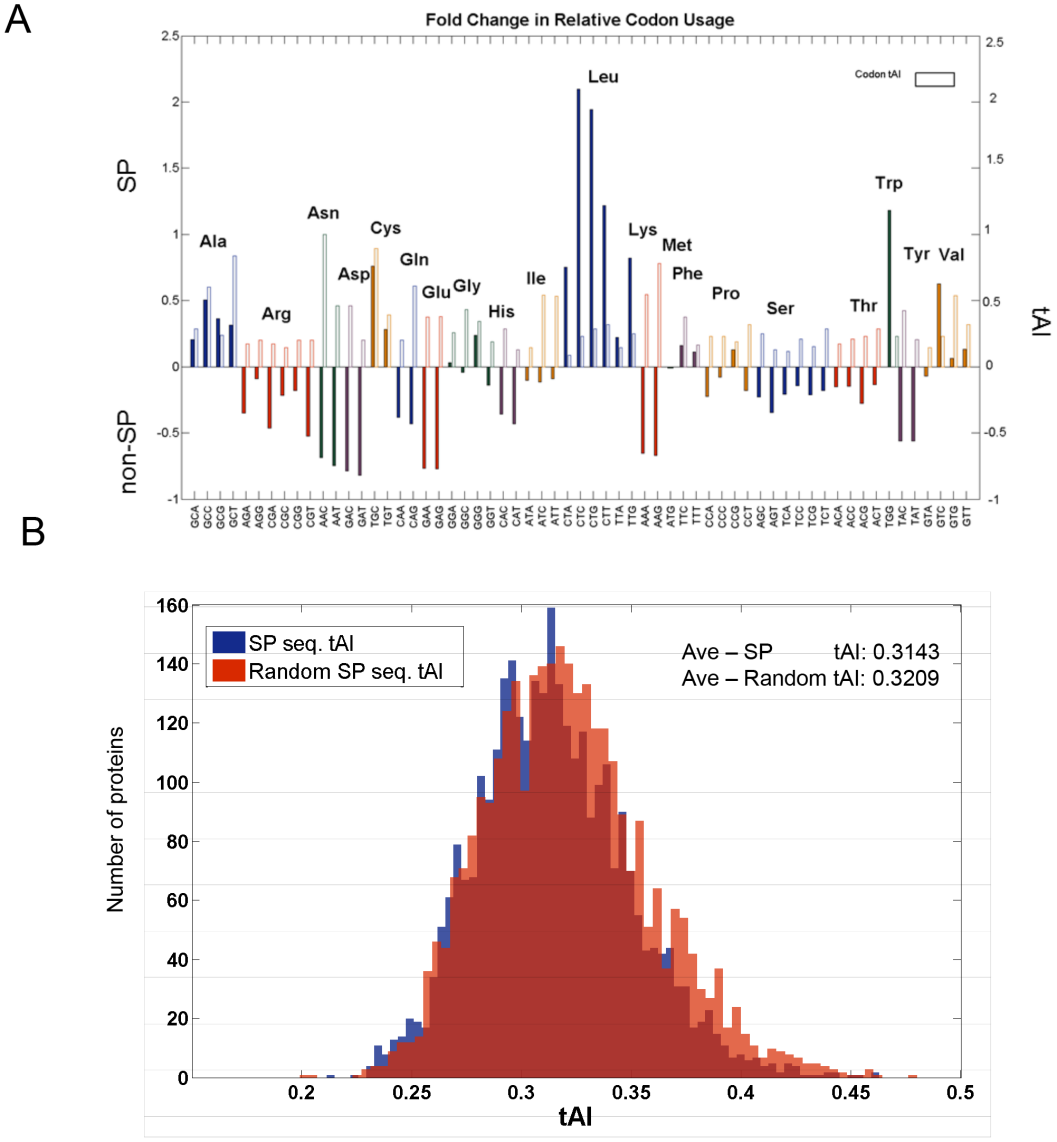
Signal sequence codon usage analysis of the human proteome. (**A**). Codon usage fold change in SP versus non-SP proteins. The relative codon usage in signal sequences is divided by the relative codon usage in sequences of same length distribution, originated from non-SP proteins. Y-axis is shown as the fold change subtracted by one marking the codons that are more commonly used and those that are underrepresented in the signal sequences. The values of codon tAI are indicated by the empty frame to indicate the absolute tAI value for each codon (as in Figure 1B). (**B**). tAI distribution of the original signal sequences (blue) and of the signal sequences in which each codon was randomly replaced by a synonymous one according to their codon usage distribution (red). The significance of the mean values of the two distributions is shown.

There are two possible explanations for the observed profile at the N1-segment of the proteins with SP sequences: (i) The AAs that determine the SP are enriched with “slower” codons (i.e., lower tAI codon values); (ii) The codons at the initial segment that compose the SP reflect an evolutionary selection process. Both explanations may fulfill the global demands of MEB-Rb translation mode. In order to distinguish between these possibilities, we counted the codon usage in the SP of each of the relevant proteins, and the codon usage in segments of non-SP proteins. For some codons, the deviation between the usage in SP and non-SP is substantial (Figure 7A). For example, the use of Cys is preferable in SP-proteins, while Lys is rarely used in the segment that covers the SP sequence. Additionally, we tested the existence of an evolutionary signal that can account for the preferential selecting of codons in the N′-terminal segments of the SP-proteome. This is performed for any AA, regardless of its actual tendency to be used. Specifically, we questioned whether a selected codon in the SP sequence is randomly chosen from a background of the complete proteome codon usage data.

We show the preferred usability of a specific codon in view of its tAI value (Figure 7A, empty frames). For example, the AA valine (Val) is encoded by four codons. Among these codons, the codons that are mostly used for the SP-proteins are the ones with low tAI values (codons GTC) while the ones with maximal tAI value (codon GTG) are rarely used (Figure 7A). In order to assess the statistical power of such observations, we compared the actual local tAI for the SP segment (as in Figure 7A) with that of simulated sequences that are composed of identical amino acids but are encoded by codons that were randomly selected from their synonymous codons, according to the tAI distribution in the entire genome (Figure 7B). While the tAI distributions are quite similar (dKL<0.001), the mean value of the actual SP local tAI value was lower with respect to the randomized sequences (0.3143 and 0.3209 for the original SP and the synonymous codons tAI 1000 randomized tests, respectively). Importantly, the distributions differ significantly from the replaced sequences according to the codon usage distribution (p-value = 1.3e-07).

We concluded that in addition to the preselected AAs for the SP sequences (Figure 7A), an evolutionary signal is attributed to the selection of preferred codons in the SP sequences (Figure 7B).

### Prototypic profiles of translational efficiency - the human proteome

**Figure 8:**
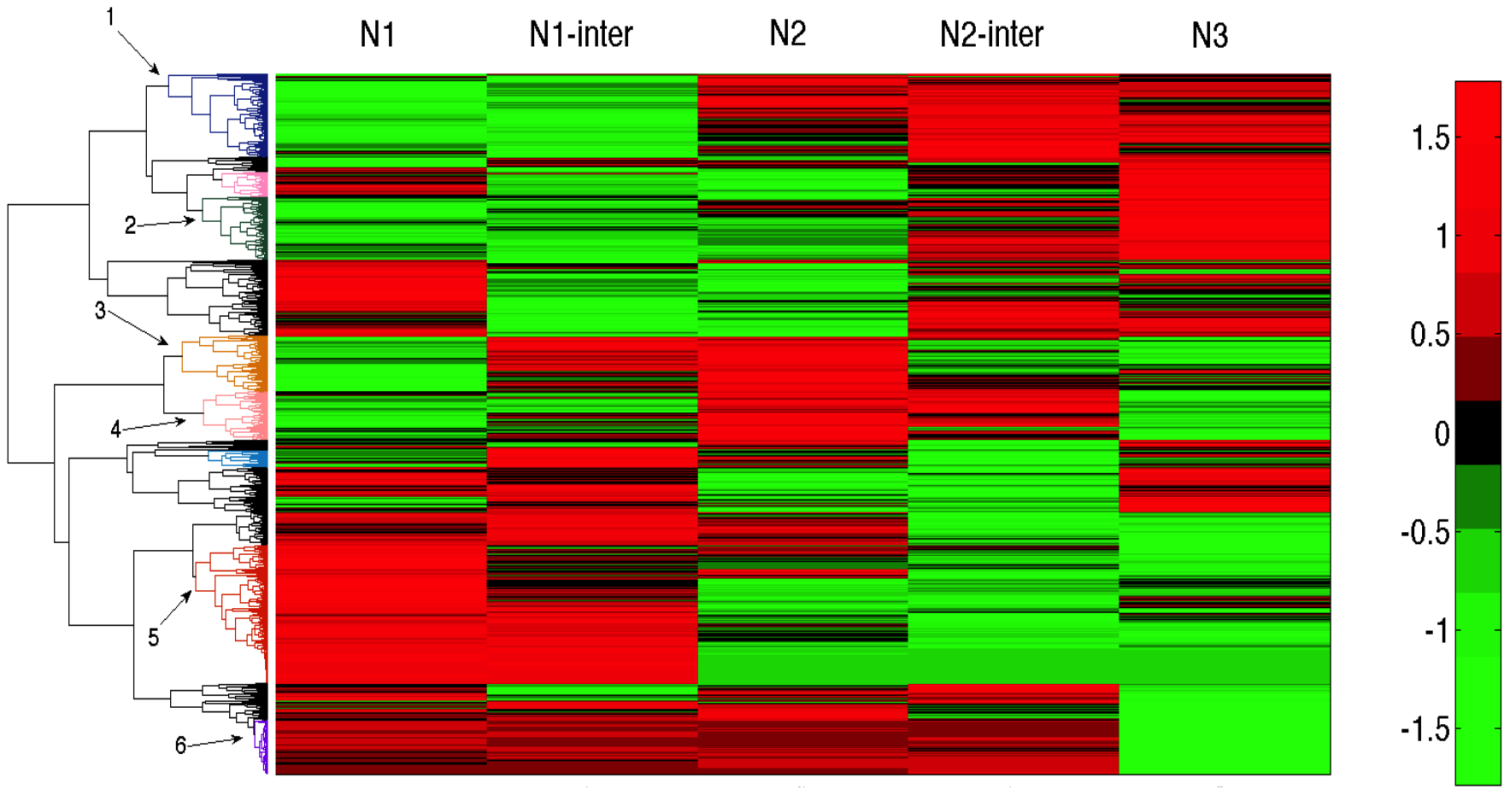
Clustering of all human proteome according to their segmental tAI values for the N′-terminal. A total of 18,434 proteins are included in the analysis and clustered by the calculated tAI for 5 consecutive overlapping segments at the N′-terminal region of the proteins. Unsupervised clustering resulted in several dominating clusters that are numbered 1–6. Red and green colors mark the low and high segmental tAI values, respectively (according to the scale). For details on annotation enrichment for each cluster, see Table S4.

The N′-terminal segmental profile of SP proteins dominated over 3,100 protein sequences in humans (Figures 4B–4G). To ensure an unbiased analysis of the human proteome, we clustered by means of an unsupervised mode all ∼18,400 human proteomes according to their segmental tAI profile (illustrated in Figure 4A). We focused on clusters that are dominated by LATS at the N1 segment (Figure 8, clusters 1–4). Enrichment tests according to the clusters' annotations were performed. The most significantly enriched cluster's annotation consists of secreted, signal, glycoprotein and disulfide-bridge (p-value of enrichment is 5.4e-18). An additional set of enriched annotations includes the plasma membrane and membranous proteins. These annotations are fully consistent with MEM–Rb translation (for a detailed analysis, see Table S4). Therefore, the clusters of most significant LATS values followed by HATS are associated with secreted proteins, membranous proteins, extracellular matrix and receptors, all of which belong to SP-containing proteins.

Based on a global, unbiased clustering, proteins that are signified by a characteristic pattern are identified. For example, a profile with several consecutive HATS (Figure 8 cluster 6,170 proteins) matches ribosomal proteins. Such a profile is expected for proteins that are expressed at high amounts and a translation speed that reaches maximal efficiency (i.e., the number of proteins that are produced per transcript). Ribosomal proteins are known by their high expression, efficient translation and the preferable use of abundant codons. A detailed analysis of proteins clusters according to the segmental tAI profile (Figure 8) is beyond the scope of this study.

## Discussion

The concept that arises from our study supports the notion of evolutionary dependent marks for a “speed control” management. We have shown that such property is encoded in the initial segment of the SP-proteins (secreted and membranous), TP-proteins (mitochondria targeted), as well as for the terminal segment of GPI-anchored proteins but not the anchor TMD sequences. Thus, the observed segmental tAI profile also acts at the level of “final destination” of proteins. The TP-proteins and the addition of the GPI-moiety [40] are post-translational processes. In the case of TP-proteins, the observed segmental tAI profile (Figure 5B) may act as a “time delayer” to ensure safe folding. Importantly, the observed signal for “speed control” management is missing for the bulk of the proteins that are translated by free ribosomes. It was proposed that the lowly adapted tRNAs at the initial segment of proteins govern the ribosomal allocation properties as expressed by ribosome density and translation speed [41]. In this report, we propose that the evolutionary encoded signal is mainly associated with membrane bound translation. We postulate that it is a general design for complying with the mechanistic and kinetic demands of a restricted subset of the proteome.

Investigating the trend of the local segmental tAI (e.g., Figures 4–5) for protein families allows us to challenge the importance of their profile in view of their function. We focused on 25 human proteins that carry Matrix Metalloproteinses (MMPs) functions [42]. This diverse group consists of membranous (6 proteins) and secreted proteins (18 proteins, Table S5). MMPs contribute to the modulation cancer and metastasis. The different MMPs regulate apoptosis, inflammation, migration, adhesion and vascularization [43]. We noted that the average local tAI profile (Figure S2) of the MMP family resembles the overall N′-terminal segmental trend of the SP-proteome (i.e., initial segment of LATS following by HATS). Interestingly, it is mostly the subset of the membranous MMPs (with/without TMD or with GPI anchor) rather than the secreted MPPs that dominates this pattern. The pattern of the local tAI and the variability in this profile among paralogs and functionally related proteins is under current investigation.

The partition of the complete proteome to four disjointed groups is based on their apparent proteins' localization. Evidently, other partitions are feasible. We tested the impact of our predetermined partition on the robustness of the observed pattern assigned for the SP-proteome: (i) We confirmed that further partition of the SP-membranous proteins to proteins with a single- or multi-TMDs (Figure 5) had no effect on the observed pattern of the entire group. (ii) The results of an unsupervised clustering procedure showed that a large fraction of the human proteome matches a small number of dominant patterns (Figure 8, Clusters 1–6). Focusing on the clusters that show a pattern similar to that of the SP-proteome revealed a significant enrichment of key terms that include ER lumen, vesicle trafficking, extracellular proteins, receptors, hormones, plasma membrane and such (Table S4). Interestingly, we identified several SP-proteins that belong to small families (e.g., defensins) that exhibit a unique tAI segmental pattern which is different from the dominant secretory clusters (clusters 1–4, Figure 8). Defensins are host-defense secreted peptides of the innate immune system. Defensins resulted from recent duplications and some were shown as specific to the primate lineage [44]. We are currently studying the translational efficiency of such outliers.

A causal relation of the tAI segmental pattern and the apparent translation efficiency is somewhat indirect (discussed in [16]). The estimation of the abundance of tRNAs *in vivo* (computationally and experimentally) showed the strong correlation to their genomic copy number [26] under a broad set of conditions. However, subtle effects of tRNA concentration at the ribosome A-site, the activity and extent of the tRNA modifying enzymes [45] and the actual fraction of the loaded/unloaded tRNAs adds to the dynamic modeling of ribosome allocation and queuing [46].

A quantitative view of the need for allocating the resources for translation was proposed based on experimental [2] and evolution considerations [3], [14]. While most of the analysis is based on *E. coli* and *S. cerevisiae* [3], the impact of the different determinants on *in vivo* translation efficiency in humans and other multicellular organisms remained an open issue [47]. The observed pattern of conserved optimal and non-optimal codons in clusters was proposed as an evolutionary evolved rhythm for the ribosomal speed in accordance with the secondary structure of the translated polypeptides [48].

Additional hardcoded signals are encoded by the CG content, the Shine-Delgarno (SD) and the Kozak sequences around the coding region's start-codon [7], [49]. Additional context-dependent features (mRNA secondary structure, RNA binding proteins, ribosomal cycle on a circular mRNA) are expected to fine-tune the *in vivo* translation efficiency.

Previous studies had not distinguished the CYTO-Rb from MEM-Rb translation [16], [50]. However, several studies support the view that ER proteins indeed impose specialized translational properties. For example, the ER-related mRNAs are long-lived [51]. Recently, using ribosomal profiling technology, the MEM-Rb fraction was compared to the CYTO-Rb fraction [13]. Striking differences were reported between the two modes of translation. Specifically, the ER fraction associates with a lower (by 2.5 fold) tendency for falling off the mRNAs (i.e., high processivity), a higher steady state loading capacity, and a significantly higher ribosomal gene density [13].

In this report, we had not explicitly elaborated on all the determinants that dominate the pattern of global (Figures 2–3) or local tAI measurements (Figures 4–6, Figure S1). We focused on some of the “hard-coded” determinants, mainly the codons and their distribution along the transcripts. A high correlation between the cellular abundance of tRNAs and the codon frequencies had been confirmed [26]. Consequently, we choose the tAI as our main measure (rather than codon usage or alternative measures). Notably, the range of tAI values for different organisms is wide (Table S1). Still, we identified a robust signal that is assigned with the N′-terminal segment of the SP-proteins in 6 different model organisms. When the same analysis was duplicated for the C′-terminal segments, there was no outstanding signal in any of these organisms (for statistical confidence see Table 1, Table S3). Recall that the analysis of the SP-proteome in human includes an average of >3,100 proteins (17% of all proteins), leading to sound statistics. Despite poor annotation coverage for some of the model organisms (excluding yeast and humans), the statistical confidence of the observed phenomena remains highly significant (Table 2).

A plausible hypothesis attributes the observed pattern of the SP-proteome to the fact that the SP sequences are composed of hydrophobic residues [52]. We argue that by using the tAI measures, the “hydrophobicity” per se cannot account for our findings: (i) The hydrophobic AAs are not particularity associated with low tAI values (Figure 1B, Table S1). (ii) The C′-terminal helical segment of the GPI-precursor lies in between the secreted SP and TMD segments in terms of hydrophobicity [53]. (iii) Despite a poor correlation of tAI values among organisms (Figure 1C), the pattern of LATS is valid for all the tested organisms (Figure 4B–4G). (iv) A component of codon selection was isolated from the impact of AA composition per se (for the 3,100 human SP-proteins). Specifically, for each AA of the SP, we replaced its codon without changing the AA identity (Figure 7B). Based on such a strict analysis, we isolated a component of codon selection. The effect is quite modest, but statistically significant (p-value = 1.3e-07). (v) Tail-anchor proteins (human, total of 639 proteins) that belong to Type IV (Figure 6) failed to show the pattern of C′-terminal LATS, despite the prominent presence of a TMD in the C′-terminal segment. (vi) The TMD from the group of “TMD not SP” showed that the hydrophobicity cannot account for low adapted codons (Figure S1).

In accordance with our view, the evolution rate for SP sequences was calculated to be 10 fold higher when compared to the mature proteins. Specifically, it was suggested that SP sequences have undergone positive selection [54]. We argue that the variability in the SP sequences is a reflection of the translation “hard-coded” speed control signals that covers these segments. Additional sequence determinants for translation efficiency include the GC content, transcript and coding length, over-representation of correlated codons [55], and the tendency for mRNA secondary structures. We showed that the GC and the coding length do not constitute the basis for our reported observations.

From an evolutionary perspective, it was proposed that an optimal strategy in enhancing translational efficiency is observed under tRNA shortage [18]. However, in addition to purely sequence-based determinants, a number of context-dependent attributes (often hard to separate) govern the translational speed *in vivo*. This includes the presentation of secondary structures, the accessibility of ribosomes and masking of the transcript by RNA binding proteins [56], [57]. Isolating these determinants is context dependent and naturally also cell specific (e.g., some cells may contain RNA binding proteins that interfere with the ribosome flow). Whether the tAI segmental profile directly governs the speed parameters for multi-cellular eukaryotes is yet to be tested.

Sophisticated imaging technologies determined the parameters of the translation elongation rate at a codon resolution [58]. In addition, *in vivo* experimental measures by ribosomal profiling [2], [13] provided detailed data on the steady state of the ribosome positioning during translation. Our current analyses provide an additional layer to the qualitative outlook of the process of elongation [59].

### Mechanistic constraints for ER bound translation

Several models were developed to capture the translation kinetics of the secretory proteome [60]–[62]. Based on this view, the signal that was exposed in this report could also serve to enhance the capacity of the mRNA to engage in a productive ER targeting process. An efficient reuse of the mRNA on MEM-Rb, once the mRNA is “occupied” by an already docked ribosome, is an attractive proposal [13], [63].

Our analysis focused on the MEM-Rb translation. We revisited the mechanistic demands of the secretory proteome [30]. In addition to the need of managing the ribosomal flow for any transcript, special constrains are imposed for the MEM-Rb translation. In mammals, the co-translocation of SP-containing proteins is mediated mostly by the signal recognition particle (SRP) [64]. Once the SRP recognizes the emerging SP from the ribosome [65], a conformational change leads to slowing of translation. Apparently, this attenuation in translation rate is necessary for the nascent chain to diffuse to the ER membrane [47]. The interaction of the SRP with its receptor (SR) and its release serve as an internal “timer” for resuming translation [66], and for production of functional proteins [67].

Recently, the SRP-independent insertion route was systematically assessed in yeast [68] and mammals [69]. The dependency of the hydrophobicity index of the N′-terminal segments of the proteins and the tendency to bind the SRP revealed that a substantial fraction of the yeast secretome is actually SRP-independent and this fraction mainly applies to SP-proteins and to the subset of the GPI proteome [68]. Thus, the notion of a “timer” for translation and translocation may not be limited to SRPs but to the need for a rich network of proteins and chaperones that coordinate their actions to ensure appropriate translocation and targeting.

A role for the codons' distribution along the transcripts as a “time delayer” should be considered. With this notion, the generality for transcripts for SP-, TP- and GPI-anchor proteins is striking. We suggest that attenuation of events such as the SP proteolytic cleavage (not necessarily in the end of the LATS), the speed of folding, the cleavage of GPI to promote the locking of the protein at the membrane surface, and recycling of the mRNA to ensure additional rounds of translation are all encoded in the codon organization profile. A similar signature across a range of organisms from yeast to humans indicates a robust, evolutionary refined phenomenon.

## Materials and Methods

### Proteins' coding sequences and experimental data

The list of proteins for each group of each organism was taken from UniProtKB based on a “reviewed” set. For SP proteins we used the UniProtKB (Based on SignalP4.0 [52]). Only proteins marked with “signal” and “cleaved site” were considered. The SP-anchored proteins were excluded from the SP-proteins group. In addition, the proteins marked as “fragment” were excluded. A similar protocol was applied for GPI-anchored and TP (transit-peptide) and predicted Tail-anchored (TA) Type IV. The canonical variants from UniProtKB were mapped to their matched RefSeq nucleotide sequences. A gene that had no matched sequence, or had a sequence that lacked the ATG initiator codon, was discarded. The corresponding coding sequences were extracted from the RefSeq database. Only proteins that start with an initiator Methionine and end with a stop codon are compiled.

Signal peptide sequences were retrieved from the proteins coding sequences according to their position that were marked by UniProtKB. The codon usage for these sequences was counted and defined as SP codon usage. The codon usage of sequence from proteins that are not annotated as SP proteins was counted as non-SP codon usage. Those sequences began at the first position of the coding sequence and terminated at a position that was randomly selected from the signal sequence length distribution. Sequences that were randomly replaced were created by replacing each codon in the sequence with a codon from its synonymous codons by a random choice according to the codon usage of each AA. Randomized tests were performed 1000 times.

A high coverage (>70%, 4,500 proteins) mass spectrometry (MS) yeast experiment [33] was used for protein abundance measurements. Protein levels span more than four orders of magnitude. Independent yeast protein quantitation was extracted from the GFP library measurements [34]. Briefly, each protein from the GFP-tagged yeast library was counted by flow cytometry measurement (∼2,500 proteins). For human protein abundance, the MS data resource for the high-coverage of 11 human cell-lines [35] was used.

### tAI measurements

An estimation of the effect of the tRNA abundance on the efficiency of the translation rate of codons is captured by the tRNA adaptation index (tAI) [19]. The tAI value for each codon is composed from two components – the amounts of the relevant tRNA and its codon–anticodon coupling. The latter is not unique - a factorization for each of the wobble pair was used [19]. Global tAI measurement gauges the availability of tRNAs for each codon along the mRNA. Data of genomic tRNA copy numbers were taken from the Genomic tRNA Database (http://gtrnadb.ucsc.edu/) using human genome hg19 (NCBI Build 37.1, Feb 2009) [70]. For each tRNA isoacceptor, the number of gene copies (excluding Pseudogenes and Selanocysteine tRNAs) was counted. The codon tAI and global tAI for the model organisms was calculated as above from Genomic tRNA Database (Table S1).

A codon–anticodon coupling is not unique - a factorization for each of the wobble pair was used [19]. Formally, let ni be the number of tRNA isoacceptors recognizing codon i. Let tCGNij be the copy number of the jth tRNA that recognizes the ith codon, and let Sij be the selective constraint on the efficiency of the codon-anticodon coupling. We have used the Sij scaling for the Wobble nucleoside-nucleoside pairing as described in [41]. We define the absolute adaptiveness, Wi, for each codon i as:

From Wi we obtain wi, which is the relative adaptiveness value of codon i, by normalizing the Wi's values (dividing them by the maximal of all the 61 Wi).

The final tAI of a gene (referred as Global tAI) is the geometric mean of its codons (excluding the stop codon). A geometric mean was calculated in an identical way for calculating the segmental tAI (e.g., 30-codons, SP-segment, TMD segment). Local tAI is calculated by dividing each coding sequence into several overlapping windows, each containing 30 codons. Relative tAI value is defined as the ratio of the segmental, local tAI (i.e., 30-codons segment) to the calculated global tAI of the protein (for the entire protein length). A relative tAI value <1.0 signifies the preference of rarely adapted tRNA codons (“slow” codons) in the analyzed segment relative to the codon composition of the entire coding sequence. Global tAI and C1 segment tAI were computed by excluding the stop codon from their sequences. For sequences that are shorter than 180 amino acids, only local segmental tAI were calculated. This was applied to avoid overlap between N′ and C′ terminal windows.

### Proteins' clustering

Protein clustering was performed for a matrix of 18,434 rows (each represents a mRNA-mapped coding sequence), and five columns (each represents a window of 30 codons from the N′-terminus segments marked N1 to N3. The functional annotation enrichment of the resulted clusters was according to Fisher Exact Test enrichment scheme with hypergeometric distribution and multiple hypothesis corrections [71].

### Statistical analysis and simulations

Different data distributions were compared using the standard Matlab statistical tools such as Kolmogorov–Smirnov (KS) and t-tests. The KS test compared any two samples while quantifying the empirical cumulative distribution functions of the two. The p-value is calculated under the null hypothesis that the samples are drawn from the same distribution. Thus, the lower p values indicate more significant differences between the two examined samples. The difference in the probability distribution between the two datasets was computed using Kullback–Leibler divergence (dKL) (see detailed in [72]). For testing the similarity of the segmental tAI profile to randomly created genes, we created random gene sets with the same codon preference and same length distribution. We selected a set of 1000 genes. The simulation was performed by 1000 repetitions of the protocol.

## Supporting Information

Figure S1 Reanalysis of the local tAI of the human “TMD not SP” proteins, according to their first TMD that serves as anchor signal.
(PDF)

Figure S2 Local tAI of 25 human Matrix metalloproteinases
(PDF)

Table S1 The tAI codons values for 6 model organisms. Each of the 61 codons are indicated by the calculated tAI. For each tRNA isoacceptor, the number of gene copies (excluding Pseudogenes and tRNA for Selanocysteine) was counted.
(DOCX)

Table S2 Partition of the complete proteomes to 4 groups.
(DOCX)

Table S3 Global tAI values for complete proteomes partitioned to 4 groups for 6 eukaryotic organisms.
(DOCX)

Table S4 Annotation enrichment summary for clusters 1–4, Figure 7.
(DOCX)

Table S5 Identifier of 25 proteins of the human Matrix metalloproteinases, input list of Figure S2.
(PDF)
