# Supplementary figures and images for: Speed Controls in Translating Secretory Proteins in Eukaryotes - an Evolutionary Perspective
Source: PLoS Comput Biol. 2014 Jan 2;10(1):e1003294. doi: 10.1371/journal.pcbi.1003294 (PMC3879104; doi:10.1371/journal.pcbi.1003294)

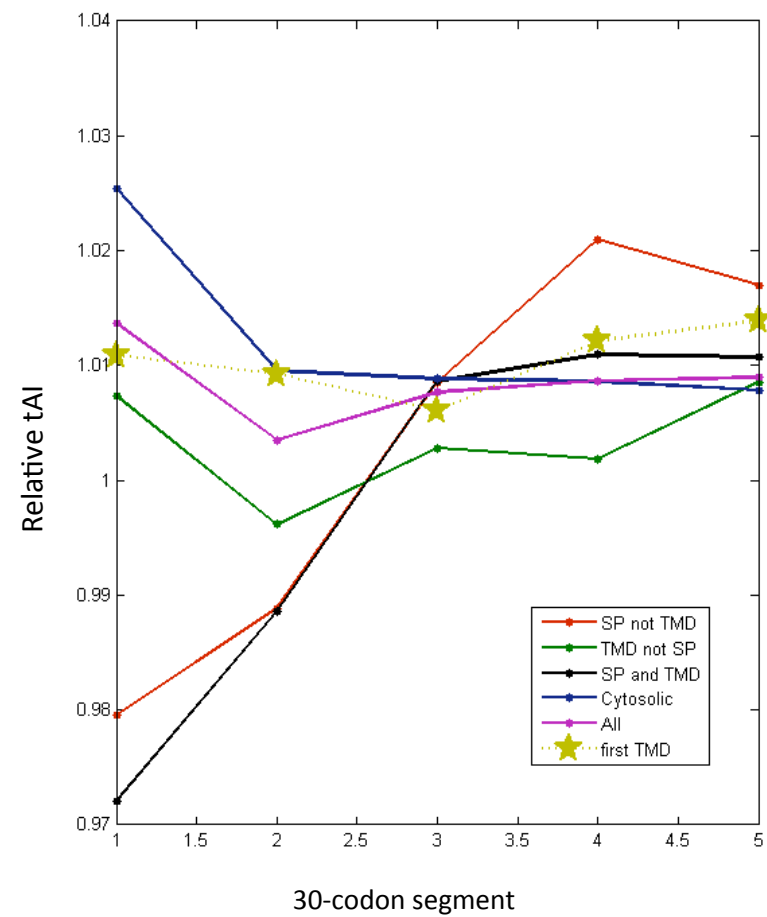

Supplement: Figure S1 — Reanalysis of the local tAI of the human “TMD not SP” proteins, according to their first TMD that serves as anchor signal. (PDF) [file pcbi.1003294.s001.pdf]

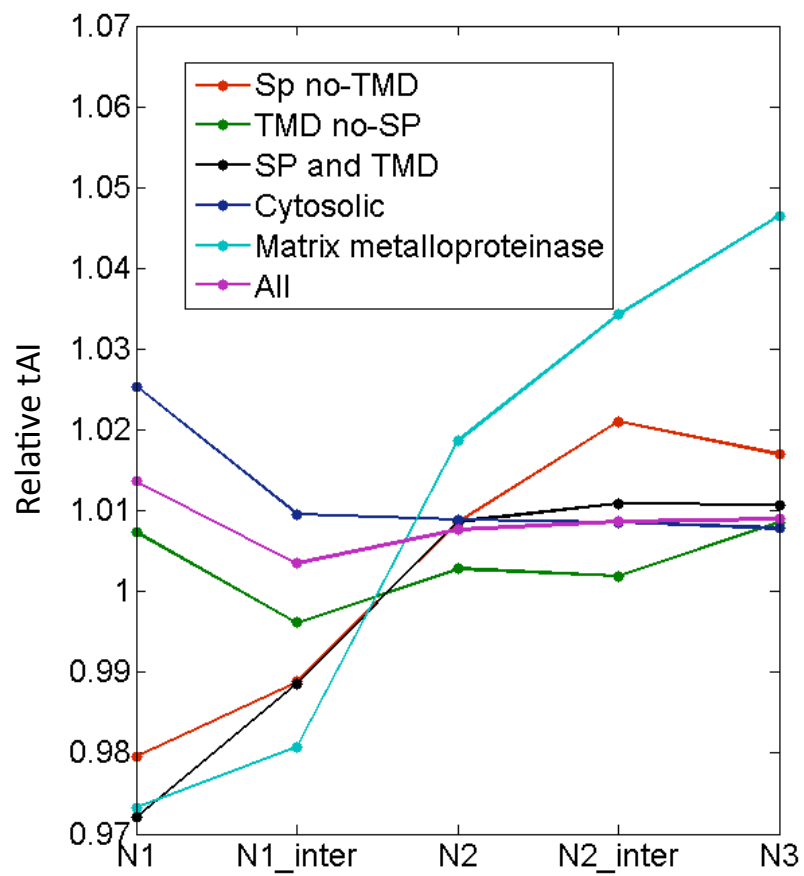

Supplement: Figure S2 — Local tAI of 25 human Matrix metalloproteinases (PDF) [file pcbi.1003294.s002.pdf]
