# Supplementary material for: Speed Controls in Translating Secretory Proteins in Eukaryotes - an Evolutionary Perspective
Source: PLoS Comput Biol. 2014 Jan 2;10(1):e1003294. doi: 10.1371/journal.pcbi.1003294 (PMC3879104; doi:10.1371/journal.pcbi.1003294)
Supplement: Table S1 — The tAI codons values for 6 model organisms. Each of the 61 codons are indicated by the calculated tAI. For each tRNA isoacceptor, the number of gene copies (excluding Pseudogenes and tRNA for Selanocysteine) was counted. (DOCX) [file pcbi.1003294.s003.docx]

**Table S1**. Codons tAI for *H. sapiens, S. cerevisiae, C. elegans, D. melanogaster, B. taurus and A. thaliana.*

| **codon** | ***H.sapiens*** | ***B.taurus*** | ***D. melanogaster*** | ***C.elegans*** | ***S.cerevisiae*** | ***A. thaliana*** |
| --- | --- | --- | --- | --- | --- | --- |
| AAA | 0.547238 | 0.061532 | 0.286807 | 0.416667 | 0.431034 | 0.173333 |
| AAC | 1 | 0.031555 | 0.573614 | 0.555556 | 0.67734 | 0.213333 |
| AAG | 0.779954 | 0.050456 | 0.713193 | 0.994444 | 1 | 0.282133 |
| AAT | 0.458698 | 0.013853 | 0.251816 | 0.243889 | 0.297352 | 0.093653 |
| ACA | 0.17284 | 0.00789 | 0.286855 | 0.250047 | 0.30795 | 0.10668 |
| ACC | 0.207373 | 0.008741 | 0.344168 | 0.34 | 0.487685 | 0.096 |
| ACG | 0.228111 | 0.007258 | 0.235182 | 0.302222 | 0.160099 | 0.1008 |
| ACT | 0.288018 | 0.01139 | 0.478011 | 0.472222 | 0.67734 | 0.133333 |
| AGA | 0.172814 | 0.032344 | 0.143403 | 0.222222 | 0.738916 | 0.12 |
| AGC | 0.251152 | 0.015683 | 0.286807 | 0.25 | 0.123153 | 0.173333 |
| AGG | 0.199309 | 0.08687 | 0.189293 | 0.21 | 0.29803 | 0.145067 |
| AGT | 0.129954 | 0.009043 | 0.125908 | 0.10975 | 0.054064 | 0.076093 |
| ATA | 0.144052 | 0.005523 | 0.09565 | 0.194506 | 0.123233 | 0.066692 |
| ATC | 0.541475 | 0.009656 | 0.344168 | 0.44 | 0.637931 | 0.1824 |
| ATG | 0.576037 | 0.026033 | 0.573614 | 0.527778 | 0.738916 | 0.32 |
| ATT | 0.53318 | 0.013411 | 0.478011 | 0.611111 | 0.827525 | 0.253333 |
| CAA | 0.201613 | 0.0142 | 0.191205 | 0.583342 | 0.554187 | 0.106667 |
| CAC | 0.288018 | 0.019059 | 0.239006 | 0.587778 | 0.492611 | 0.133333 |
| CAG | 0.611751 | 0.032943 | 0.443595 | 0.381111 | 0.238916 | 0.154133 |
| CAT | 0.12644 | 0.009985 | 0.104924 | 0.315028 | 0.216256 | 0.058533 |
| CCA | 0.230446 | 0.007101 | 0.239039 | 0.972239 | 0.615776 | 0.600023 |
| CCC | 0.228111 | 0.006816 | 0.240918 | 0.12 | 0.08867 | 0.1632 |
| CCG | 0.18894 | 0.006216 | 0.315488 | 0.422222 | 0.197044 | 0.258667 |
| CCT | 0.31682 | 0.009466 | 0.334608 | 0.166667 | 0.123153 | 0.226667 |
| CGA | 0.172831 | 0.009468 | 0.478059 | 0.305608 | 4.31E-05 | 0.080012 |
| CGC | 0.145161 | 0.015052 | 0.344168 | 0.38 | 0.310345 | 0.0864 |
| CGG | 0.199309 | 0.013285 | 0.152964 | 0.153333 | 0.061576 | 0.092267 |
| CGT | 0.201613 | 0.014161 | 0.478011 | 0.527778 | 0.431034 | 0.12 |
| CTA | 0.086437 | 0.003945 | 0.095626 | 0.083383 | 0.184729 | 0.133349 |
| CTC | 0.228111 | 0.00568 | 0.172084 | 0.36 | 0.061576 | 0.128533 |
| CTG | 0.286866 | 0.008362 | 0.413002 | 0.165556 | 0.059113 | 0.082667 |
| CTT | 0.31682 | 0.007889 | 0.239006 | 0.5 | 0.027032 | 0.165853 |
| GAA | 0.374424 | 0.32738 | 0.286807 | 0.472222 | 0.923645 | 0.16 |
| GAC | 0.460829 | 0.039696 | 0.669216 | 0.75 | 0.923645 | 0.346667 |
| GAG | 0.379032 | 0.220725 | 1 | 0.817778 | 0.418719 | 0.224533 |
| GAT | 0.202304 | 0.020664 | 0.293786 | 0.32925 | 0.40548 | 0.152187 |
| GCA | 0.288102 | 0.022091 | 0.09566 | 0.250061 | 0.30795 | 0.133355 |
| GCC | 0.601382 | 0.018617 | 0.413002 | 0.44 | 0.487685 | 0.1536 |
| GCG | 0.236175 | 0.01969 | 0.173996 | 0.191111 | 0.098522 | 0.136 |
| GCT | 0.835253 | 0.024359 | 0.573614 | 0.611111 | 0.67734 | 0.213333 |
| GGA | 0.259217 | 0.277683 | 0.286807 | 1 | 0.246305 | 0.16 |
| GGC | 0.432028 | 0.053832 | 0.669216 | 0.416667 | 0.985222 | 0.306667 |
| GGG | 0.342166 | 1 | 0.091778 | 0.403333 | 0.20197 | 0.117867 |
| GGT | 0.18966 | 0.032804 | 0.293786 | 0.182917 | 0.432512 | 0.134627 |
| GTA | 0.144041 | 0.017357 | 0.095631 | 0.138942 | 0.184815 | 0.093353 |
| GTC | 0.228111 | 0.013064 | 0.206501 | 0.38 | 0.62069 | 0.144 |
| GTG | 0.535714 | 0.039475 | 0.365201 | 0.211111 | 0.182266 | 0.136533 |
| GTT | 0.31682 | 0.018144 | 0.286807 | 0.527778 | 0.862069 | 0.2 |
| TAA | 0.028805 | 0.003156 | 0.262268 | 0.30313 | 0.061576 | 0.157197 |
| TAC | 0.423963 | 0.034079 | 0.43021 | 0.527778 | 0.492611 | 1 |
| TAG | 0.009217 | 0.009687 | 0.262268 | 0.027778 | 0.019704 | 0.157197 |
| TAT | 0.205818 | 0.020356 | 0.188862 | 0.231694 | 0.216256 | 0.439 |
| TCA | 0.115236 | 0.003945 | 0.095641 | 0.250042 | 0.246373 | 0.120049 |
| TCC | 0.207373 | 0.031618 | 0.275335 | 0.3 | 0.487685 | 0.368533 |
| TCG | 0.152074 | 0.006784 | 0.221797 | 0.357778 | 0.140394 | 0.091733 |
| TCT | 0.288018 | 0.021434 | 0.382409 | 0.416667 | 0.67734 | 0.499187 |
| TGA | 0.086406 | 0.019734 | 0.047801 | 0.027778 | 0.061576 | 0.157197 |
| TGC | 0.892857 | 0.356473 | 0.334608 | 0.361111 | 0.246305 | 0.2 |
| TGG | 0.229263 | 0.130952 | 0.397706 | 0.342222 | 0.389163 | 0.186667 |
| TGT | 0.391964 | 0.239578 | 0.146893 | 0.158528 | 0.108128 | 0.0878 |
| TTA | 0.144009 | 0.006311 | 0.191205 | 0.138889 | 0.492611 | 0.08 |
| TTC | 0.374424 | 0.024928 | 0.382409 | 0.416667 | 0.67734 | 0.213333 |
| TTG | 0.247696 | 0.013853 | 0.25239 | 0.238889 | 0.773399 | 0.158933 |
| TTT | 0.164372 | 0.013641 | 0.167878 | 0.182917 | 0.297352 | 0.093653 |
