# Supplementary material for: Speed Controls in Translating Secretory Proteins in Eukaryotes - an Evolutionary Perspective
Source: PLoS Comput Biol. 2014 Jan 2;10(1):e1003294. doi: 10.1371/journal.pcbi.1003294 (PMC3879104; doi:10.1371/journal.pcbi.1003294)
Supplement: Table S2 — Partition of the complete proteomes to 4 groups. (DOCX) [file pcbi.1003294.s004.docx]

**Table S2.** Number of proteins according to 4 distinct groups partition in six different organisms.

| **Organism** | **SP non-TMD** | **TMD non-SP** | **non-SP non-TMD** | **SP and TMD** |
| --- | --- | --- | --- | --- |
| *H. sapiens* | 1758 | 3395 | 11887 | 1394 |
| *B. taurus* | 636 | 926 | 3655 | 265 |
| *D. melanogaster* | 302 | 475 | 2198 | 119 |
| *C. elegans* | 296 | 542 | 2243 | 106 |
| *A. thaliana* | 1258 | 1868 | 7084 | 423 |
| *S. cerevisiae* | 215 | 1074 | 4658 | 93 |
