# Supplementary material for: Speed Controls in Translating Secretory Proteins in Eukaryotes - an Evolutionary Perspective
Source: PLoS Comput Biol. 2014 Jan 2;10(1):e1003294. doi: 10.1371/journal.pcbi.1003294 (PMC3879104; doi:10.1371/journal.pcbi.1003294)
Supplement: Table S3 — Global tAI values for complete proteomes partitioned to 4 groups for 6 eukaryotic organisms. (DOCX) [file pcbi.1003294.s005.docx]

|  | *H.*  *sapiens* | *B. taurus* | *D. melanogaster* | | *C.*  *elegans* | *A.*  *thaliana* | *S.*  *cerevisiae* |
| --- | --- | --- | --- | --- | --- | --- | --- |
| SP no-TMD | 0.3246 | 0.0268 | 0.3352 | 0.4101 | | 0.1705 | 0.4206 |
| TMD no-SP | 0.3208 | 0.0244 | 0.3192 | 0.3646 | | 0.1686 | 0.3696 |
| SP and TMD | 0.3201 | 0.0253 | 0.3202 | 0.3703 | | 0.1666 | 0.3663 |
| Cytosolic | 0.3224 | 0.0270 | 0.3415 | 0.4004 | | 0.1701 | 0.3883 |

**Table S3.** Global tAI values for the proteins according to 4 distinct groups in 6 different organisms.
