# Supplementary material for: Speed Controls in Translating Secretory Proteins in Eukaryotes - an Evolutionary Perspective
Source: PLoS Comput Biol. 2014 Jan 2;10(1):e1003294. doi: 10.1371/journal.pcbi.1003294 (PMC3879104; doi:10.1371/journal.pcbi.1003294)
Supplement: Table S4 — Annotation enrichment summary for clusters 1–4, Figure 7. (DOCX) [file pcbi.1003294.s006.docx]

**Table S4.** Functional enrichments resulted for the hierarchical clustering of the human proteome. The clustering is based on the vector of 5 consecutive tAI segments values (covers 90 codons from the N’-terminal region) and 18,434 protein sequences.

| Cluster | Number of  proteins | Dominating InterPro and GO terms | C-EASE score^a^ | Most significant  p-value | Most significant FDR |
| --- | --- | --- | --- | --- | --- |
| 1,3 | 2160 | Glycoprotein, signal peptide, disulfide bond, Secreted, extracellular region | 9.995 | 5.38e-18 | 8.14e-15 |
| 1 | 2160 | Cell membrane, plasma membrane, transmembrane region, intrinsic to membrane | 7.731 | 1.15e-05 | 13.10 |
| 1 | 1527 | Oxidoreductase, electron carrier activity, oxidation reduction | 3.605 | 5.66e-04 | 0.93 |
| 2 | 1162 | Extracellular matrix, extracellular region | 4.078 | 6.81e-5 | 0.10 |
| 2 | 1629 | Receptor,signal peptide, g-protein coupled receptor, Olfactory receptor, G protein-coupled olfactory receptor, class II, sensory transduction | 2.904 | 1.11e-6 |  |
| 2 | 1162 | Transit peptide, mitochondrion | 2.454 | 1.50e-5 | 0.028 |
| 4 | 1154 | Gap junction, cysteine-rich domain, Connexins, wide pore channel activity, cell-cell junction | 5.034 | 3.76e-7 | 6.29e-4 |

^a^C-EASE score is roughly the average enrichment for all keywords in the cluster transformed to -log10 units.
