# Supplementary material for: Speed Controls in Translating Secretory Proteins in Eukaryotes - an Evolutionary Perspective
Source: PLoS Comput Biol. 2014 Jan 2;10(1):e1003294. doi: 10.1371/journal.pcbi.1003294 (PMC3879104; doi:10.1371/journal.pcbi.1003294)
Supplement: Table S5 — Identifier of 25 proteins of the human Matrix metalloproteinases, input list of Figure S2. (PDF) [file pcbi.1003294.s007.pdf]

Table S5. The UniProtKB identifiers list the human Matrix metalloproteinase family.

Entry	Entry name	Localization
P35613	BASI_HUMAN	SP, TMD
P09238	MMP10_HUMAN	SP
P24347	MMP11_HUMAN	SP
P39900	MMP12_HUMAN	SP
P45452	MMP13_HUMAN	SP
P50281	MMP14_HUMAN	SP, TMD
P51511	MMP15_HUMAN	SP, TMD
P51512	MMP16_HUMAN	SP, TMD
Q9ULZ9	MMP17_HUMAN	SP, GPI
Q99542	MMP19_HUMAN	SP
P03956	MMP1_HUMAN	SP
O60882	MMP20_HUMAN	SP
Q8N119	MMP21_HUMAN	SP
O75900	MMP23_HUMAN	TMD, Signal-Anchor
Q9Y5R2	MMP24_HUMAN	SP, TMD
Q9NPA2	MMP25_HUMAN	SP, GPI
Q9NRE1	MMP26_HUMAN	SP
Q9H306	MMP27_HUMAN	SP
Q9H239	MMP28_HUMAN	SP
P08253	MMP2_HUMAN	SP
P08254	MMP3_HUMAN	SP
P09237	MMP7_HUMAN	SP
P22894	MMP8_HUMAN	SP
P14780	MMP9_HUMAN	SP
Q9BV57	MTND_HUMAN	
